# Genetic association of *ADIPOQ* gene variants (-3971A>G and +276G>T) with obesity and metabolic syndrome in North Indian Punjabi population

**DOI:** 10.1371/journal.pone.0204502

**Published:** 2018-09-28

**Authors:** Harjit Kaur, Badaruddoza Badaruddoza, Veena Bains, Anupam Kaur

**Affiliations:** Department of Human Genetics, Guru Nanak Dev University, Amritsar, Punjab, India; CHA University, REPUBLIC OF KOREA

## Abstract

**Background and aims:**

At present obesity and metabolic syndrome (MetS) in India are the most challenging health problems. It is also well accepted that obesity is a significant risk factor for the development of metabolic syndrome and other degenerative diseases. Many studies have reported that single-nucleotide polymorphisms (SNPs) of the adiponectin (*ADIPOQ)* gene have been associated with obesity and its related disorders. Here, we aimed to investigate the association of two intronic variants in *ADIPOQ* gene, -3971A>G (rs822396) and +276G>T (rs1501299) with obesity and metabolic syndrome.

**Methods:**

Biochemical and anthropometric measurements were obtained from a total of 550 unrelated subjects (obese = 250 and non-obese = 300) of North Indian Punjabi population. Genotyping for the intron variants were performed by polymerase chain reaction based restriction fragment length polymorphism (PCR-RFLP) methods. After genotyping, as a quality control measure 10% of the samples for each polymorphism were confirmed by Sanger Sequencing method. The distributions of genotypic and allelic frequencies among obese and non-obese groups were compared by chi-square test and the corresponding risk was calculated using binary logistic regression. The effects of multiple testing were controlled by applying Bonferroni corrections.

**Results:**

All the anthropometric and biochemical parameters except triglycerides (TG) and very low-density lipoproteins cholesterol (VLDL-C) have shown significant association with both GG and TT genotypes of -3971A>G (rs822396) and +276G>T (rs1501299) polymorphisms respectively. The frequencies of GG (-3971A>G) and TT (+276G>T) genotypes were higher among obese cases (p = 0.008 and p = 0.035 respectively). However, no significant association was found between allelic frequencies of *ADIPOQ* rs822396 and obesity, whereas the association of *ADIPOQ* rs1501299 attenuated and became marginally significant after Bonferroni correction (p>0.025). Both the variant genotypes of *ADIPOQ* gene polymorphisms (-3971GG and +276TT) significantly increased the risk of development of obesity (OR: 1.52, p = 0.03; OR: 1.58, p = 0.04 respectively) and MetS (OR: 1.42, p = 0.03; OR: 1.57, p = 0.01 respectively) in the present population, after adjusting the various covariates. The G-T haplotype model (possessing -3971G and +276T alleles) was shown toprovide ~ 3 fold risk towards the obesity susceptibility (OR: 2.69, p = 0.009) and MetS risk (OR: 2.73, p = 0.009) and the association persisted after adjusting for different confounding variables.

**Conclusion:**

The present study has confirmed that *ADIPOQ* -3971A>G (rs822396) and +276G>T (rs1501299) polymorphism may be clinically helpful to estimate obesity and MetS risk in North Indian Punjabi population.

## Introduction

At present obesity and metabolic syndrome (MetS) in India are the most challenging health problems with tremendous increase in the last decade [1,2]. These two health problems are significantly associated with anthropometeric, physiometeric and Biochemical parameters such as body mass index (BMI), waist circumference (WC), waist to hip ratio (WHR), systolic blood pressure(SBP), diastolic blood pressure (DBP), TG and HDL [3–8]. Obesity has a very complex pathogenesis with reference to genetic, environmental and lifestyle factors. These parameters directly interfere and control the total body metabolism with the help of different biological molecules such as leptin, adiponectin, adipsin, interleukin 6 etc. Among them, adiponectin plays an important role in the regulation of energy homeostasis, carbohydrate metabolism and insulin sensitivity [9–11].

Adiponectin (*ADIPOQ*) is the most abundant gene product in the adipose tissue which is encoded by adipocyte, C1q, and collagen domain containing (ACDC) [12]. The *ADIPOQ* gene spans 16kb consisting of three exons and two introns on chromosome 3q27. The region of adiponectin gene has been investigated by several investigators in different population in the search for genetic variants that are significantly related with the pathophysiology of obesity, metabolic syndrome (MetS), diabetes and associated complications[1,12–17]. Moreover, serum adiponectin concentrations are also highly heritable and are linked to *ADIPOQ* gene [18,19], underlining the relevance of studying *ADIPOQ* as the candidate gene for obesity and consequently MetS.

The individuals of Indian subcontinent have specific phenotype which is characterized by increased abdominal obesity despite low body mass index (BMI), hyperinsulinemia [16,20] and dyslipidemia [21]. It has been reported from previous many studies that the measurement of waist circumferences] (WC) is more accurate measure of the distribution of body fat to define central obesity especially in Asian Indians. However, epidemiological surveys used body mass index (BMI) as an indicator of generalized obesity due to its simplicity, in terms of ease to measure weight and height. Therefore, BMI still prevails as a surrogate measure of adiposity worldwide and is the most widely accepted definition of obesity. Both generalized and central obesity have been associated with metabolic abnormalities [16, 22–24]. Despite the fact, BMI cut-off points are used clinically to identify high-risk individuals for screening and absolute risk assessment.

A number of population based studies has related an association between single nucleotide polymorphisms (SNPs) in *ADIPOQ* gene with obesity, insulin resistance, MetS and circulating levels of adiponectin [25–27] and have reported conflicting evidence in different populations. The polymorphisms selected for the present study at the *ADIPOQ* locus are A to G substitution in intron 1(-3971 A>G, rs822396) and G to T substitution in intron 2 (+276G>T, rs1501299). Several human studies have been done to find out the association between +276 G>T (rs1501299) polymorphism and obesity in different ethnic populations. In many studies, the investigator have proved strong association of this polymorphism with obesity and associated risk [1,12,16,19,26,28,29]. Whereas, many other studies have failed to detect any association [30–34]creating controversies as to whether this polymorphism is correlated with obesity and suggested genetics and heterogeneity or population/ethnicity specific association.

The inconsistency in association of -3971 A>G (rs822396) polymorphism with obesity was reported for various populations [16,33,35,36], however, the positive associations were replicated in some. No systematic analysis of the -3971 A>G (rs822396) polymorphism with regard to obesity and associated risk was previously reported from North Indian population. The lack of GWAS from India is due to negligible consortiums and limited infrastructure [37]. However, a GWAS study from Japan have showed positive association of rs822396 polymorphism with confectionery intake (P = 0.049 for stage 2 and 4.2x 10^−5^ for stage 1+2), implying that this polymorphism appears to be biologically credible as an indicator of a gene involved in the development of obesity [36]. It has been suggested that variants in promoter region of *ADIPOQ* gene are associated with, either independently or as a part of haplotype with T2DM and BMI [38–41]. Recently, a researcher have reported strong association between -3971A>G (rs822396) polymorphism with obesity in South Indian populations [16]. To our knowledge, there is no published study regarding association of these adiponectin polymorphisms from North Indian Punjabi population.

Therefore, on the basis of conflicting results regarding the association of adiponectin polymorphism with obesity and metabolic syndrome in different populations [12,26,2,29,31,42–45], the present investigation selected the two variants of *ADIPOQ* gene; -3971 A>G (rs822396) and +276 G>T (rs1501299) respectively, to investigate their putative association with obesity and metabolic syndrome in North Indian Punjabi population.

## Material and methods

### Study subjects

The present case control study included a total of 550 unrelated adult subjects recruited from different districts of Punjab, a North Indian state. There were 250 obese cases with BMI >25 kg/m^2^ (mean age 37.85±9.767 years and mean BMI 32.25±4.31 kg/m^2^) and 300 non obese healthy controls with BMI<23 kg/m^2^ (mean age 38.10±10.92 years and mean BMI 21.24±1.27 kg/m^2^). Individuals with heavy muscles, cardiovascular disease, endocrine disorders, liver or kidney diseases, confirmed diabetes, malignancies, pregnancy and any other chronic disease were not included in the present study. All the study subjects were interviewed through a questionnaire to provide information regarding their socio-demographic characteristics, personal history, presence of disease, drug intake, diet and lifestyle patterns. A written informed consent was also obtained from all the participants. The study was approved by Ethical Committee constituted by Guru Nanak Dev University, Amritsar, Punjab.

### Anthropometric and physiometric parameters

Anthropometric measurements, such as height, weight, waist circumference (WC), and hip circumference (HC) were taken in a standardized manner. Weight and height of individuals were measured to the nearest 0.1kg and 0.5cm respectively with light clothing and without shoes. Waist circumference was measured to the nearest 0.1cm and hip circumference was measured at the widest point of the hip with the subject standing with both the feet together. The body mass index (BMI) was calculated as the weight in kg divided by square of height in meters. Waist to hip ratio (WHR) was calculated as waist circumference (cm) divided by hip circumference (cm). Systolic and diastolic blood pressure (SBP and DBP) was measured after 5 min reset period by using mercury sphygmomanometer. Sedentary lifestyle was defined as one who daily invests <n minutes in leisure activities(n = 25minutes/day in women and 30 minutes/day in men[46] The subjects were classified as vegetarian and non-vegetarian according to their food habits.

### Definition of risk factors

Generalized obesity was defined as BMI ≥25 according to WHO 2000 criteria for Asian [47, 48]. Metabolic syndrome was defined using diagnostic criteria given by international diabetes federation 2005[49], according to which an individual with central obesity (defined as WC >80 cm for women and WC > 85 cm for men) and any two or more following risk factors was considered as having MetS: increased TG(>150 mg/dl), reduced HDL-C(<40 mg/dL in men, <50 mg/dL in women), elevated blood pressure (SBP≥130 mmHg or DBP ≥ 85 mmHg) and increased fasting glucose (≥ 100 mg/dL).

### Biochemical analysis

Overnight fasting blood samples were collected from each participant to measure serum lipids, total cholesterol (TC), triglycerides (TG), high-density lipopoprotein (HDL) cholesterol using kits provided by supplier [Erba-Mannheim (Transasia Bio-medicals Ltd., Solan, India)]. Low-density lipoprotein (LDL) and very low-density lipoprotein (VLDL) were calculated by fridewald’s formula [50].

### DNA analysis

Genomic DNA was extracted from peripheral blood leucocytes pellet using Phenol/Chloroform method and after that, diluted to a working concentration of 20 ng/ml for further use and stored at -20°C until use for genotyping. The genotyping of polymorphism *ADIPOQ* -3971 A>G (rs822396) and +276 G>T (1501299) were performed by polymerase chain reaction based Restriction Fragment Length Polymorphism (PCR-RFLP) methods. Amplification of DNA sample was done by using following flanking primers: F: 5′-CAGGCTGATCGCACCTATTA-3'and R: 5′-CATCCTTTTCTGCTGGGAAA-3' for (-3971 A>G), F: 5′-GTC TCT CCA TGG CTG ACA GT-3' and 5′- GGT GAA GAT GGG AAA GGG GA-3' (reverse) for +276 G>T [16, 51].

The PCR procedure was performed with 15μL reaction mixture (50ng of template DNA, 4μM of each primer, 200μM of each dNTP, 0.3 U/ml of Taq Polymerase and 1X Pcr buffer, NEB) consisted of an initial melting step of 10 min at 94° C followed by 30 cycles of 45sec at 94° C, 45sec at 58° C(for +276G>T), 45sec at 60° C(for -3971A>G), and 45sec at 72°C and final elongation step of 10 min at 72°C.

**Fig 1:**
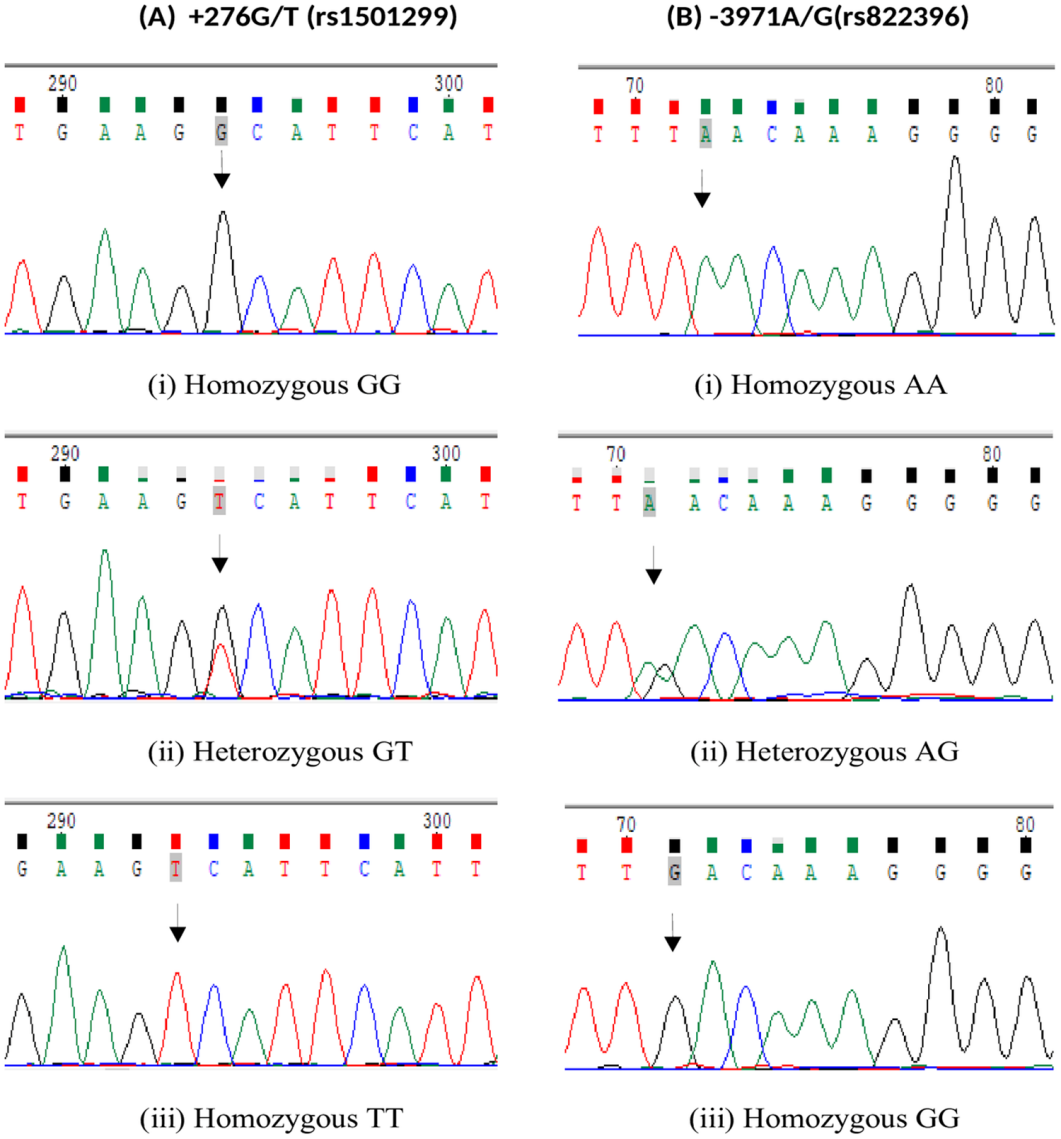
Sequencing chromatograms demonstrating the homozygous wild type, hetrozygous and homozygous mutant genotypes for (A) +276 G>T (rs1501299) and (B) -3971 A>G (rs822396) SNPs.

PCR products (246bp for -3971 A>G and 504bp for 276G>T) were detected on 2% agarose gel stained with ethidium bromide (EtBr), amplified PCR products were digested with 5U of restriction enzymes MseI and BsmI (New England Biolabs, USA) for -3971A>G (rs822396) and +276G>T (rs1501299) respectively, for 12–16 hrs at 37° C with 1x NEB buffer4 in a final volume of 15 μL reaction followed by heat inactivation for 20min at 65°C. The digestion resulted in fragments of 147bp and 99bp for A allele (-3971A>G) and 321bp and 183bp for G allele (+276G>T). The homozygous mutant allele remains undigested. The restriction products were resolved by Agarose gel electrophoresis (2.5% for -3971A/G and 3% for +276G/T) stained with ethidium bromide (10mg/ml)(GeNei^™^) against 100 bp ladder as standard and viewed under UV transilluminator. For each reaction, a negative control (without DNA template) was added to monitor PCR contamination. After genotyping, as a quality control measure 10% of the samples for each polymorphism were confirmed by Sanger Sequencing method (Fig 1).

### Statistical analysis

Statistical analyses were performed using SPSS Version 21.0. The clinical parameters were represented as means ± standard deviation and were compared by the students’t- test. The chi-square test (χ^2^) was used to compare the genotype or allele frequencies. We used SNPstats to test the genotype frequencies for the Hardy-Weinberg equilibrium and to determine the haplotype frequencies, odds ratios (ORs), two tailed p-values and 95% confidence interval (CI) were calculated as the measure of association of the SNPs and the presence of obesity and metabolic syndrome. Binary logistic regression analysis was used to identify the risk of different genotypes of selected polymorphism for obesity and MetS and the values were adjusted for possible confounding effect of age, gender, diet and lifestyle patterns. A p-value <0.05 was considered statistically significant for all the tests. Significant p-values obtained were corrected for multiple testing using Bonferroni correction.

## Results

### Patient characteristics

The clinical and biological characteristics of subjects are summarized in Table 1. There were no significant differences in HDL-C concentration, pulse pressure, TG, VLDL-C and diet pattern between obese and non-obese groups. The prevalence of metabolic syndrome and sedentary lifestyle were significantly higher (p<0.001) in obese than in the non- obese groups. As compared to non-obese individuals, the obese subjects had significantly higher mean value for BMI, WC, WHR, WHtR, SBP, DBP, fasting glucose level, TC and LDL-C.

**Table 1:** Clinical and biochemical characteristics of the study population.

Variables	Non-obese (n = 300)	Obese (n = 250)	p values
Age(years)	38.13±10.40	37.85±9.767	0.001*
Sex-ratio (men/women)	0.304	0.179	0.022
Height(cm)	161.13±8.90	158.16±9.15	0.038
Weight(kg)	55.29±7.00	80.68±12.72	0.001*
BMI(kg/m^2^)	21.24±1.27	32.25±4.31	0.001*
WC(cm)	78.07±9.31	105.59±9.32	0.001*
WHR	0.82±0.09	0.94±0.07	0.001*
WHtR	0.49±0.06	0.67±0.06	0.001*
SBP(mm Hg)	118.64±13.66	128.79±14.99	0.001*
DBP(mm Hg)	76.32±11.44	83.27±10.84	0.001*
Pulse rate(mm Hg)	79.43±10.36	83.08±10.25	0.026
Pulse pressure	42.32±10.33	45.52±12.88	0.09
Fasting glucose(mg/dl)	79.51±14.71	86.87±14.67	0.002*
Met S*n* (%)	34 (11.30	73 (31.2)	0.001*
TC(mg/dl)	164.84±28.83	185.19±37.19	0.001*
TG(mg/dl)	123.88±63.00	141.99±70.46	0.05
HDL-C(mg/dl)	43.79±12.02	42.78±10.90	0.574
LDL-C(mg/dl)	95.03±26.16	114.01±39.90	0.001*
VLDL-C(mg/dl)	24.77±12.60	28.39±14.09	0.05
Sedentary life style *n* (%)	65 (21.67	187 (74.8	0.001*
Active life style *n* (%)	235 (78.33)	63 (25.2)	
Vegetarian *n* (%)	191 (63.67)	171 (68.4)	0.281
Non-vegetarian *n* (%)	109 (36.33)	79 (31.6)	

Mean ± standard deviation or n (%). BMI, body mass index; WC, waist circumference; WHR, waist to hip ratio; WHtR, waist to height ratio; SBP, systolic blood pressure; DBP, diastolic blood pressure; MetS, metabolic syndrome; TC, total cholesterol; TG, triglyceride; HDL-C, high density lipoprotein-cholesterol; LDL-C, low density lipoprotein cholesterol; VLDL-C, very low density lipoprotein cholesterol.

*p< 0.002 is statistically significant with Bonferroni correction

### Genotype frequencies

The genotype distributions of *ADIPOQ*-3971A>G (rs822396) and +276G>T (rs1501299) polymorphisms in the combined study population, obese cases and non-obese controls are presented in Table 2. The frequency of GG genotype of -3971 A>G (rs822396)polymorphism was significantly higher in the obese group(12.4%) than in the non-obese group(6%) group (p<0.008). The frequencies of AA and AG genotypes were found to be higher among the non-obese group (45.6% vs 49.3% for AA and 42.0% vs 44.67% for AG respectively between obese and non-obese groups), but the difference was not significant. A statistically significant difference in the genotype frequencies of the-3971A>G polymorphism among obese and non obese group was found to be diminished after correcting for multiple testing (Bonferroni threshold p<0.025). Similarly, the frequency of mutant TT genotype of +276G>T (rs1501299)*ADIPOQ* gene polymorphism was also higher among the obese subjects (17.6%) as compared to control group (11.3%) but was not statistically significant, as the resulted p value was greater than Bonferroni threshold of 0.025 (0.05/2). Whereas, the frequencies of GG and GT genotypes were higher in non-obese group but were statistically non-significant. There was no significant association between the allele frequencies of -3971A>G (rs822396) polymorphism and obesity whereas in context to +276G>T (rs1501299) polymorphism, a marginal association (p = 0.02) persisted at allelic level after Bonferroni correction.

**Table 2:** Genotype frequencies of *ADIPOQ*-3971A>G (rs822396)and +276G>T (rs1501299) in obese, non-obese and combined study population.

*ADIPOQ*SNP’s	Genotypes/Alleles	Study Population %(n) (550)	Obese%(n) (250)	Non-obese%(n) (300)	p^a^-value	p^b^-value
-3971A>G (rs822396)	AA	47.64(262)	45.6(114)	49.3(148)	0.383	0.032
	AG	43.46(239)	42.0(105)	44.67(134)	0.532	
	GG	8.9(49)	12.4(31)	6.00(18)	**0.008**	
	A	69.37(763)	66.6(333)	71.64(430)	------	0.06
	G	30.63(337)	33.4(167)	28.34(170)	------	
+276G>T (rs1501299)	GG	59.09(325)	56(140)	61.67(185)	0.178	0.103
	GT	26.73(147)	26.4(66)	27(81)	0.862	
	TT	14.18(78)	17.6(44)	11.33(34)	0.035	
	G	72.45(797)	69.2(346)	75.17(451)	------	**0.02**
	T	27.54(303)	30.8(154)	24.83(149)	-------	

^a^Comparison between obese and non obese subjects for each genotype;

^b^Comparison between obese and non obese subjects for overall genotypes; Bonferroni corrected p = 0.025 (The significance level p = 0.05 is divided by number of selected SNP’s); SNPs, single nucleotide polymorphisms.

### LD estimation between *ADIPOQ* polymorphisms and haplotype analysis

**Fig 2:**
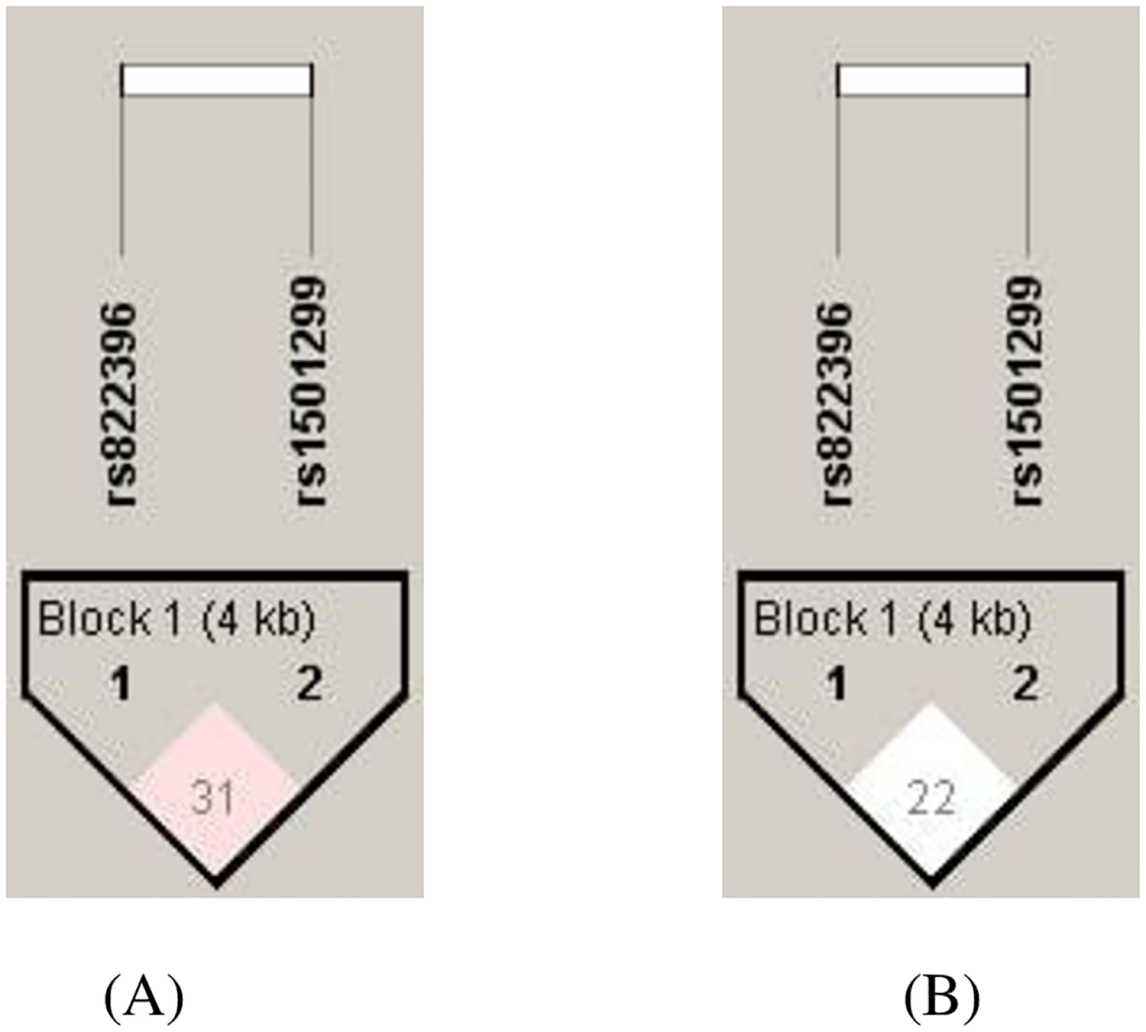
LD plot showing the position of the two *ADIPOQ* polymorphisms and pair-wise D’ values observed in the study population with respect to (A) obesity (B) MetS.

The distribution of haplotype frequency and measure of Linkage disequilibrium (LD) for the study population is represented in Table 3. Based on the measures LD, it could be inferred that the two studied SNPs of *ADIPOQ* (rs822396A>G and rs1501299 G>T) were in very low LD among both the obesity (D’ = 0.319; r^2^ = 0.018) and metabolic syndrome (D’ = 0.22; r^2^ = 0.028) risk (Fig 2). The GT haplotype consisting of two mutated alleles-3971G and +276T was significantly higher (p = 0.003) in obese group than those in non-obese group and it was shown to confer3.5 fold risk towards the obesity susceptibility (p = 0.002).

**Table 3:** Distribution of haplotypes frequency and measure of LD, observed in comparison of *ADIPOQ* SNPs in combined study population.

Haplotype-3971A>G/+276G>T	Study Population %(n)(550)	Obese %(n) (250)	Non-obese%(n) (300)	OR(CI)(p-values)	p^a^-value	p^b^-value
AG	0.462(254)	0.416(104)	0.5(150)	Reference	0.049	0.015
GG	0.255(140)	0.264(66)	0.247(74)	1.28(0.84–1.94(p = 0.23)	0.639	
AT	0.227(125)	0.232(58)	0.223(67)	1.24(0.81–1.92)(p = 0.31)	0.806	
GT	0.056(31)	0.088(22)	0.03(9)	3.52(1.56–7.96)**(p = 0.002)**	**0.003**	
LD measure	Obesity			Metabolic syndrome		
-3971A>G and+276G>T	D’	r^2^		D’	r^2^	
	0.319	0.018		0.22	0.028	

^a^Comparison between obese and non obese subjects for each genotype;

^b^Comparison between obese and non obese subjects for overall genotypes; Bonferroni corrected p = 0.0125 (The significance level p = 0.05 is divided by number of haplotypes); Order of SNPs in *ADIPOQ* haplotypes: -3971A>G, +276G>T.

### Characteristics of study population according to genotypes

The clinical characteristics of the study population across the genotypes have been summarized in Table 4. The minor allele GG of -3971A>G (rs822396) polymorphism was significantly associated with higher mean values of BMI, TC, HDL-C, LDL-C, WC, WHR and WHtR compared to other genotypes. Similarly, the TT variant genotype of +276G>T (rs1501299) polymorphism revealed significant association with increasing BMI, WC, WHR, WHtR and elevated levels of TC and TG in comparison to the other genotypes. All the significant associations persisted after Bonferroni correction (p<0.05).

**Table 4:** Clinical characteristics of the study population according to *ADIPOQ*-3971A>G(rs822396) and +276G>T (rs1501299) genotypes.

SNP’s Parameters	-3971A/G (rs822396)			+276G>T (rs1501299)			P- values
	AA	GA	GG	GG	GT	TT	
BMI (kg/m^2^)	28.76±6.28	27.04±6.55	30.29±3.86§	27.96±5.01	23.76±3.79	28.99±3.21§	0.015^a^ 0.001^b^
TC (mg/dl)	162.32±20.26	173.50±34.28†	179.81±26.51†	165.17±25.11	178.00±26.42†	202.79±24.50†	0.004^a^ 0.001^b^
TG (mg/dl)	133.02±49.87	122.89±47.89	137.44±38.36	116.29±41.11	129.79±45.55	164.53±33.09§	0.317^a^ 0.001^b^
HDL-C (mg/dl)	47.03±10.27	42.97±10.68†	43.46±8.65	37.97±9.26	44.16±10.56†	32.16±4.89	0.048^a^ 0.001^b^
LDL-C (mg/dl)	106.48±28.29	102.81±31.03	132.02±21.12§†	106.09±37.73	109.15±37.81	154.36±20.75†	0.001^a^ 0.001^b^
VLDL-C (mg/dl)	29.07±14.44	25.69±12.20	28.99±18.89	24.04±8.66	28.14±14.17	30.74±9.57	0.283^a^ 0.153^b^
WC (cm)	94.09±12.98	92.37±17.27	105.68±10.56§†	85.84±7.15	89.80±13.30	97.10±10.91§†	0.001^a^ 0.006^b^
WHR	0.86±0.12	0.87±0.09	0.91±0.98†	0.89±0.08	0.88±0.09	0.95±0.06§†	0.013^a^ 0.013^b^
WHtR	0.61±0.11	0.58±0.11	0.69±0.09§†	0.62±0.11	0.62±0.09	0.73±0.11§†	0.001^a^ 0.001^b^

^a^Comparison for -3971A>G (rs822396);

^b^Comparison for +276G>T (rs1501299)^,^

^†^p>0.05 compared with wild type;

^§^p>0.05 compared with heterozygote, tested by ANOVA with the Bonferroni method.

### Association between *ADIPOQ* polymorphisms with obesity and MetS risk

Table 5 represents the associations of *ADIPOQ*SNPs -3971A>G (rs822396) and +276G>T (rs1501299) with metabolic syndrome risk. The GG and TT genotypes of -3971A>G and +276 G>T polymorphisms respectively have shown significant association with metabolic syndrome risk [OR: 1.45(1.15–1.54); p = 0.03 and 1.65(1.19–1.95); p = 0.04 respectively] after adjusting for various covariates such as age, gender, diet pattern and lifestyle factors. It has also been observed that the GG genotype of rs822396 (-3971A>G) polymorphism and TT genotype of rs1501299 (+276G>T) polymorphism has provided significant risk towards the obesity susceptibility [OR:1.52 (0.98–2.16), p = 0.03 and OR: 1.58(0.93–1.99); p = 0.04 respectively] and this association also persisted after adjustment to age, gender, diet pattern and lifestyle factors (Table 6).

**Table 5:** Association between *ADIPOQ* polymorphisms and metabolic syndrome risk.

SNP’s	Genotypes	Unadjusted OR(CI) (p-value)	Adjusted OR^1^ (p-value)	Adjusted OR^2^ (p-value)
**-3971A>G(rs822396)**	AG/AA	1.15(1.02–1.28) p = 0.19	1.18(1.04–1.29) p = 0.12	1.16(1.03–1.28) p = 0.18
	GG/AA	1.42(1.15–1.54) **p = 0.03**	1.47(1.14–1.54) **p = 0.01**	1.45(1.15–1.54) **p = 0.03**
**+276G>T(rs1501299)**	GT/GG	1.21(1.03–1.39) p = 0.19	1.26(1.02–1.39) p = 0.26	1.23(1.03–1.40) p = 0.25
	TT/GG	1.57(1.26–1.89) **p = 0.01**	1.63(1.20–1.88) **p = 0.04**	1.65(1.19–1.94)**p = 0.04**

OR^1^ = After adjustments for age, gender and diet pattern; OR^2^ = After adjustmentsfor age, gender, diet pattern and life style; OR, odds ratios; CI, confidence interval

**Table 6:** Association between *ADIPOQ* polymorphisms and obesity risk.

SNP’s	Genotypes	Unadjusted OR(CI) (p-value)	Adjusted OR^1^ (p-value)	Adjusted OR^2^ (p-value)
**-3971A>G(rs822396)**	AG/AA	0.95(0.91–1.08) p = 0.09	0.96(0.92–1.19) p = 0.21	0.95(0.90–1.07) p = 0.08
	GG/AA	1.52(0.98–2.16) **p = 0.03**	1.44(0.97–1.96) p = 0.05	1.46(0.97–1.98) **p = 0.03**
**+276G>T(rs1501299)**	GT/GG	0.81(0.73–0.89) p = 0.11	0.83 (0.74–0.90) p = 0.12	0.82 (0.73–0.90) p = 0.12
	TT/GG	1.58(0.93–1.99) **p = 0.04**	1.68(0.85–2.05) **p = 0.04**	1.65(0.85–1.99)**p = 0.03**

OR^1^ = Afteradjustments for age, gender and diet pattern;

OR^2^ = After adjustments for age, gender, diet pattern and life style.

### Association of *ADIPOQ* haplotypes with obesity and MetS risk

It seems in the analysis of the haplotypes of the two polymorphisms that GT haplotype possessing -3971G and +276T alleles tends to increase the risk of obesity and metabolic syndrome (OR: 2.69; p = 0.009 and 2.73; p = 0.001) respectively after adjusting the confounding variables (Table 7).

**Table 7:** Association between *ADIPOQ* polymorphisms haplotypes, obesity and metabolic syndrome risk in study population.

Obesity risk				MetS risk		
Genotypes	Adjusted OR*	CI	p-value	Adjusted OR*	CI	p-value
GG/AG	1.12	0.73–1.69	0.603	2.11	0.75–5.94	0.152
AT/AG	1.14	0.73–1.76	0.559	0.775	0.366–1.64	0.507
GT/AG	2.69	1.25–5.78	**0.009**	2.73	0.735–4.76	**0.001**

*After adjustments for age, gender, diet pattern and life style

### Comparison of minor allele frequency in different study population

A comparison of minor allele frequencies (MAF) of *ADIPOQ* -3971A>G (rs822396) and +276 G>T (rs1501299) polymorphisms in different study populations has been presented in Table 8. The discrepancy in minor allele frequencies were observed for both the studied SNPs.

**Table 8:** Comparison of minor allele frequencies (MAF) of *ADIPOQ* -3971A>G(rs822396) and +276 G>T (rs1501299)polymorphisms in different study populations.

Population	MAF (%)	Disease status	Association Status	References
**-3971A>G(rs822396)**				
**Punjab**	**33.4**	**Obesity, MetS**	**Present**	**Present study**
South Indian(Chennai)	35.6	Obesity,T2DM	Present	Ramya et al. [16]
Tunisian	18	T2DM	Present	Mtiraoui et al. [52]
Latvian	22	T2DM	Absent	Kalnina et al. [33]
German	25.4	Disinhibition	Present	Rohde et al.[53]
Korean	9.14	Ischemic stroke	Present	Cheong et al., [54]
**+276G>T(rs1501299)**				
**Punjab**	**27.54**	**Obesity, MetS**	**Present**	**Present study**
North Indian(Uttar Pradesh)	10.94	Insulin resistance syndrome	Present	Prakash et al. [55]
Tunisian	30.15	Obesity, MetS	Present	Boumaizaet al.[14]
Egyptian	76.4	Obesity	Present	Zaki et al. [12]
South-Indian (Chennai)	17.7	Obesity	Present	Ramya et al. [16]
Chinese	20.06	T2DM	Absent	Toy et al. [56]
Italian	29.9	Obesity, insulin resistance syndrome	Present	Menzaghi et al. [21]
Latavian	0.28	Obesity,T2DM	Absent	Kalnina et al. [33]
Danish	29.59	Obesity	Absent	Tanko et al. [32]
Korean	28.3	T2DM	Present	Kang et al. [57]
Chinese	26.7	MetS	Absent	Li et al. [34]
Japanese	23.2	T2DM	Present	Hara et al. [40]
Prague	28	Obesity	Present	Krizova et al. [58]
France	29	Obesity	Present	Bouatia-Najiet al.[59]
Taiwanese	30.55	Obesity, MetS, T2DM	Present	Yang et al. [60]
Austrian	30	MetS	Present	Heid et al. [19]

## Discussion

The obesity and metabolic syndrome are the established risk factors to develop cardiovascular and other degenerative diseases which are the most important causes of morbidity and mortality [14,16,61,62]. The association of genetic variations in the adiponectin gene with various obesity measures and metabolic syndrome has been extensively examined in many ethnic populations [1, 14, 16,21,40,42,60,63,64,65–68] and established the adiponectin gene as a known marker of adiposity in humans [66]. The present study is the first one to investigate the potential association between -3971A>G (rs822396) polymorphism with obesity and metabolic syndrome in North Indian Punjabi population which has not been observed in previous studies [40,69]. In this cohort, a statistically significant difference was observed in the genotype distribution of -3971A>G (rs822396) among obese cases and controls (p = 0.032) and there were significantly more carriers of GG genotypes in the obese group (12.4%) than in non-obese group (6%). It is also revealed that rs822396 variant genotype contributes 1.45 folds (p = 0.03) higher risk towards metabolic syndrome and 1.46 times (p = 0.03) increased susceptibility to obesity in the study subjects of North India. Our findings are consistent with a study conducted by Ramya et al. [16] revealing the novel association of the -3971A>G (rs822396) polymorphism with the generalized obesity in the south Indians These effects could be related to the fact that as an intronic variant, -3971A>G (rs822396) might affect the splicing of mRNA’s and leads to the formation of unstable or unprocessed mRNA’s which further impairs the adiponectin gene function. Qiao et al [70] has also proposed that the regulatory elements present at the first intron of the *ADIPOQ* gene are extremely sensitive to the adipogenic transcription factor that confers significant activity on the *ADIPOQ* gene promoter. Presence of a 34bp enhancer in the first intron was also shown to regulate the expression of adiponectin gene and it can be advised that -3971A>G (rs822396) variant present close to this enhancer is of prime importance.

While examining the association between +276G>T (rs1501299) variant and obesity, it was observed that the minor allele (TT) conferred 1.65 folds higher risk towards susceptibility to obesity and metabolic syndrome in the present population. The frequency of TT genotype was found to be significantly higher among the obese group (17.6%) as compared to non-obese group (11.3%). Our findings are in agreement with the data presented by Jang et al.[71] who reported that the phenotypic effects of the minor allele ‘TT’ of rs1501299 polymorphism were more prevalent among the subjects with increased BMI values. *ADIPOQ* rs1501299 polymorphism was found to have a significant relationship with obesity and obesity-related traits in several studies in African, Asian, American and European populations among children, adults and older adults [12,13,17,28,72,73]. However, in Tunisian population +276 G>T (rs1501299) variant was shown to provide protection towards obesity risk contradicting the present study [14]. A number of other genetic association studies of this SNP with obesity attributes have also revealed somewhat inconsistent findings in different study groups thus implying the requirement for replication studies in different ethnic populations [33,32,34]. These differences may be attributed to ethnic heterogeneity and population diversity [74]. Additionally, genetic studies alone are not sufficient enough to discover the involved SNP’s, especially in a polymorphic gene with most of the SNP’s in LD.

The present study has revealed a statistically significant association of both the mutated genotypes -3971GG (rs822396) and +276 TT (rs1501299) with different obesity parameters (BMI, WC, WHR and WHtR) suggesting the possible implication of these biomarkers in obesity and increased abdominal fat. The present association study suggests that the carriers *ADIPOQ* gene variants could be at a greater risk of general and regional fat deposition leading to increased metabolic abnormalities especially among Punjabi population. Our results are in consistent with the study conducted on Egyptian young adults where significant relationship were revealed between the rs1501299 and various obesity related traits including BMI, waist to hip ratio and mid upper arm circumference[12]. Similar results were observed in South Indian population [16], whereas, Ogundele et al. [75] has reported absence of significant association between +276 G>T (rs1501299) and obesity measures in Nigerian population. Our study also reports a significant association of -3971GG and +276 TT genotypes with metabolic syndrome risk factors (TC, HDL and LDL-C). In accordance to present study, Bertheir et al. [30] has shown that plasma adiponectin concentrations and lipoprotein/lipid levels are influenced by the +276 G>T (rs1501299) polymorphism and further concluded that effect of these adiponectin gene polymorphisms are thought to be modulated by the presence of visceral obesity. However, this study does not measure the adiponectin levels but it does establish a significant relationship between the rs1501299 and abdominal obesity measures such as WC and WHtR. It might be suggested that the variant genotypes could lead to impaired transport of triglycerides and/or cholesterol. It has also been proposed that +276 G>T variant might exist in strong linkage disequilibrium with other variants in this intronic region of *ADIPOQ* gene which could lead to unstable pre-mRNA and further results in reduced mRNA levels [65] or a presence of linkage disequilibrium with a gene in close proximity could lead to consequently higher plasma lipid levels. It is still a debate to unravel the possible role of a silent polymorphism on lipoprotein concentrations and further research in this aspect could lead to better understanding of involved pathophysiological mechanisms.

In our haplotype analysis, the frequency of GT haplotype was found to be significantly higher among the obese subjects and the association remained significant after multiple correction (Bonferroni correction). The association analysis have shown that the GT haplotype has contributed 2.69 (p = 0.009) and 2.73 (p = 0.009) times increased risk towards the development of generalized obesity and metabolic syndrome risk respectively in the study subjects. These significant associations were persisted after the adjustments to age, gender, diet pattern and lifestyle factors.

The present case control study has some strengths and limitations. To our best knowledge, this is the first North Indian study that provides the data on the genetic association between *ADIPOQ* variants and susceptibility towards obesity and metabolic syndrome risk. The study collected the sufficient data regarding the diet patterns and lifestyle behaviors which could act as potential confounding factors in the disease development. These variables can be further involved in determining the gene-environment interactions. One limitation of the present study was that, for reasons of cost- effectiveness, all the SNP’s with confirmed association reported in the literature were not genotyped. The SNP’s for the present study were simply selected on the basis of information revealed from literature review and their higher allele frequency in SNP database.

In conclusion, this study has provided significant evidence that *ADIPOQ* -3971A>G (rs822396) and +276G>T (rs1501299) polymorphisms and their haplotype combinations in North Indian Punjabi population were significantly associated with obesity risk and metabolic syndrome parameters. In the view of gene-gene interactions, further research is necessary to ascertain the significant implication of *ADIPOQ* gene variants towards the development of obesity and other features of metabolic disorders to elucidate the pathological processes.
